# Supplementary material for: Synthetic nanobody–SARS-CoV-2 receptor-binding domain structures identify distinct epitopes
Source: bioRxiv. 2021 Jan 27:2021.01.27.428466. Preprint. [Version 1] doi: 10.1101/2021.01.27.428466 (PMC7852268; doi:10.1101/2021.01.27.428466)
Supplement: 1 [file NIHPP2021.01.27.428466-supplement-1.pdf]

bioRxiv preprint doi: https://doi.org/10.1101/2021.01.27.428466; this version posted January 27, 2021. The copyright holder for this preprint (which was not certified by peer review) is the author/funder. This article is a US Government work. It is not subject to copyright under 17 USC 105 and is also made available for use under a CC0 license.

Supplementary Materials for

Synthetic nanobody–SARS-CoV-2 receptor-binding domain structures identify distinct epitopes

Javeed Ahmad[1†], Jiansheng Jiang[1†], Lisa F. Boyd[1], Kannan Natarajan[1],

David H. Margulies[1*]

Correspondence to: dmargulies@niaid.nih.gov (D.H.M)

This PDF file includes:

Materials and Methods

Supplementary Text

Figs. S1 to S8

Tables S1 to S3

bioRxiv preprint doi: https://doi.org/10.1101/2021.01.27.428466; this version posted January 27, 2021. The copyright holder for this preprint (which was not certified by peer review) is the author/funder. This article is a US Government work. It is not subject to copyright under 17 USC 105 and is also made available for use under a CC0 license.

Materials and Methods

Subcloning, expression and purification of RBD, spike, and sybody proteins

The sequences encoding the RBD of the SARS-CoV-2 spike protein (amino acids 333 to 529) were subcloned into pET21b(+), (Novagen) via *Nde*I and *Eco*RI restriction sites, using pcDNA3-SARS-CoV-2-RBD-8his (Addgene #145145, (*33*)) as template. The primers used were forward primer, 5'-TGCAGTCATATGAATCTTTGTCCGTTCGGTGAG and reverse primer, 5'-TGCAGTGAATTCTCACCCTTTTTGGGCCCACAAACT. The RBD was expressed as inclusion bodies in *E. coli* strain BL21(DE3) (Novagen). Expression, isolation of inclusion bodies, denaturation and reduction was done in 6 M guanidine hydrochloride and 0.1 mM DTT as described elsewhere (*34*). Briefly, refolding was carried out in a refolding buffer supplemented with oxidized and reduced glutathione and arginine for 3 days at 4 °C followed by dialysis against HEPES buffer (25 mM HEPES, pH 7.3, 150 mM NaCl). Concentrated and filtered protein was analyzed by size-exclusion chromatography on a Superdex 200 10/300 GL column (GE Healthcare) equilibrated with HEPES buffer. The peak corresponding to 24 kDa (monomeric) protein was collected, concentrated and further purified by ion-exchange chromatography on Mono-Q® (Cytiva).

Plasmids pSb-init encoding sybodies Sb16, Sb45. and Sb68 (Addgene #153524, #153526, and #153527, respectively) were originally reported by Walter et al (*35*) and generously made available. All plasmids were verified by DNA sequencing. Purification of the recombinant proteins from the periplasm of *E. coli* MC1061 was based on a protocol described elsewhere (*36*). Briefly, *E. coli* MC1061, transformed with a sybody-encoding plasmid, was grown in Terrific Broth (TB) medium (Gibco) supplemented with 25 μg/ml chloramphenicol, at 37 °C with shaking at 160 rpm for 2 hrs. The temperature was then decreased to 22 °C until A_{600}

bioRxiv preprint doi: https://doi.org/10.1101/2021.01.27.428466; this version posted January 27, 2021. The copyright holder for this preprint (which was not certified by peer review) is the author/funder. This article is a US Government work. It is not subject to copyright under 17 USC 105 and is also made available for use under a CC0 license.

reached 0.5. Protein expression was induced by addition of L-(+)-arabinose (Sigma) to a final concentration of 0.02% (w/v) and expression continued overnight at 22 °C and 160 rpm. The next day cells were collected by centrifugation at 2000 x *g* for 15 minutes. The cell pellet was then washed twice in PBS and resuspended in periplasmic extraction buffer (50 mM Tris/HCl pH 8.0, 0.5 mM EDTA, 0.5 μg/ml lysozyme, 20% w/v sucrose (Sigma)) at 4 °C for 30 min followed by addition of TBS (pH 8.0) and 1 mM $MgCl_2$. Cells were then centrifuged at 10,000 rpm (Fiberlite™ F21-8 x 507 Fixed Angle Rotor) for 30 min. Following transfer of the supernatant to a fresh tube, imidazole was added to a final concentration of 10 mM. Ni-NTA resin (Qiagen) equilibrated with TBS was added to the supernatant and incubated for 1 hr at RT with mild agitation. The resin was collected, washed three times with buffer supplemented with 30 mM imidazole and sybody proteins were eluted with 300 mM imidazole in TBS.

Plasmid encoding spike HexaPro (designated "S" throughout) was procured from Addgene (#154754) and transfected into Expi293F™ cells (ThermoFisher Scientific) using manufacturer's protocol. Briefly, Expi293F™ cells were seeded to a final density of 2.5-3 × 10^6 viable cells/ml and grown overnight at 37 °C in Expi293™ Expression Medium (Gibco). The following day, cell viability was determined, and cell density was adjusted to 3 × 10^6 viable cells/ml with fresh, prewarmed Expi293™ Expression Medium. Transfection was then done as per manufacturer's instructions using 1 μg/ml plasmid DNA. Cultures were grown for 6 days following transfection and supernatant was collected, filtered through a 0.22 μm filter and passed over Ni-NTA resin for affinity purification. Further purification was accomplished by size-exclusion chromatography using a Superose 6 10/300 GL column (Cytiva) in a buffer consisting of 2 mM Tris pH 8.0, 200 mM NaCl.

bioRxiv preprint doi: https://doi.org/10.1101/2021.01.27.428466; this version posted January 27, 2021. The copyright holder for this preprint (which was not certified by peer review) is the author/funder. This article is a US Government work. It is not subject to copyright under 17 USC 105 and is also made available for use under a CC0 license.

Preparative and analytical size-exclusion chromatography

Sybodies purified by Ni-NTA affinity chromatography were concentrated using Amicon 10K MWCO concentrators and purified on a Sepax SRT-10C SEC100 column at a flow rate of 1 ml/min. Monomeric sybodies elute at a retention volume of 11–12.5 ml from the Sepax SRT-10C SEC100 column. Monomeric peak fractions were collected and analyzed by SDS-PAGE. Analytical SEC of RBD sybody complexes was performed on a Shodex KW-802.5 column at a flow rate of 0.75 ml/min in TBS buffer (pH. 8.0). (The interaction of individual sybodies with the column matrix is a well-documented phenomenon (*36*)).

Surface Plasmon Resonance

SPR experiments were performed on a Biacore T200 (Cytiva) at 25 °C in 10 mM HEPES pH 7.2, 150 mM NaCl, 3 mM EDTA, 0.05% Tween-20. RBD was immobilized on a series S CM5 sensor chip (Cytiva) by amine (NHS/EDC) coupling to flow cells. For background subtraction a reference cell was mock coupled. Binding and kinetic studies were performed multiple times for each sybody. Graded and increasing concentrations of SB16, SB45 and SB68 were injected over the RBD-immobilized surface at a flow rate of 30 μl/min with an association time of 120 s and dissociation time of 2000 s. Binding data were analyzed by surface site affinity distribution analysis by EVILFIT (*37, 38*). In general, these values were consistent with fits to the Langmuir binding equation for a 1:1 interaction model using Biacore T200 Evaluation Software v3.0, but revealed better statistics.

Thermal stability

bioRxiv preprint doi: https://doi.org/10.1101/2021.01.27.428466; this version posted January 27, 2021. The copyright holder for this preprint (which was not certified by peer review) is the author/funder. This article is a US Government work. It is not subject to copyright under 17 USC 105 and is also made available for use under a CC0 license.

Thermal melt analysis of the recombinant proteins was performed in triplicate in 96-well plates in a QuantStudio 7 Flex real time PCR machine (Applied Biosystems). Each well contained 2-4 μg protein in buffer (25mM TRIS pH 8, 150 mM NaCl) and 5x Sypro Orange (Invitrogen, stock 5000x) in a total volume of 20 μl. Following an initial two-minute hold at 25 ^{o}C, the plate was heated to 99 ^{o}C at a rate of 0.05 ^{o}C/sec. Data were analyzed with Protein Thermal Shift Software v1.3 (Invitrogen) to obtain T_m values for RBD, S, Sb16, Sb45, and Sb68 (Figure S8).

Crystallization, data collection, structure determination and crystallographic refinement

Purified sybodies (Sb16, Sb45 and Sb68) and RBD were mixed in approximate 1:1 molar ratio to a final concentration of 8 mg/ml. The protein mixtures were incubated on ice for 1 hour prior to screening. Initial screening for crystals was carried out using the hanging drop vapor diffusion method using the Mosquito robotic system (sptlabtech.com). Crystals of SB16-RBD and SB45-RBD complexes and Sb16 alone were observed within one week using Protein Complex (Qiagen) and Wizard Classic 4 (Rigaku). Conditions for Sb16–RBD were either 0.1M HEPES pH 7.0, 15% PEG 20000, or 0.1M HEPES pH 7.0, 18% PEG 12000; and for Sb45–RBD was 18% PEG 12000 and 12% PEG 8000, 0.1 M HEPES pH 7.5, 0.2 M NaCl. Sb16 alone crystallized in 20% PEG 4000, 0.1 M MES pH 6.0, 0.2 M $LiSO_4$. We also screened mixtures of two or three sybodies with RBD. Crystals of Sb45–RBD–Sb68 were obtained after one month following mixing the three proteins in an equimolar ratio in 10% PEG 8000, 0.1M sodium cacodylate pH 6.0.

Crystals of protein complexes were optimized with slight adjustments of the concentration of PEG components. Crystals were cryoprotected in mother liquor containing 5%

bioRxiv preprint doi: https://doi.org/10.1101/2021.01.27.428466; this version posted January 27, 2021. The copyright holder for this preprint (which was not certified by peer review) is the author/funder. This article is a US Government work. It is not subject to copyright under 17 USC 105 and is also made available for use under a CC0 license.

ethylene glycol and 5% glycerol and flash frozen in liquid nitrogen for data collection. Diffraction data were collected at the Southeast Regional Collaborative Access Team (SER-CAT) beamline 22ID-D at the Advanced Photon Source, Argonne National Laboratory and data were processed with XDS (*39*). Multiple data sets were collected for the protein complexes from 2.3-3.2 Å resolution. The initial model of Sb16 and Sb45 for the molecular replacement search were built by the MMM server (manaslu.fiserlab.org/MMM (*40*)), using the heavy chain V domain and the RBD of the Fab B38–RBD complex (PDBid: 7BZ5) (*41*). The initial model of Sb68 for molecular replacement was built based on the V_H domain of 7BZ5. Molecular replacement solutions were found using Phaser (*42, 43*). Subsequent refinements were carried out with PHENIX (*44*). CDR loops were manually rebuilt by fitting to the electron density maps with Coot (*45*). In particular, Sb68 CDR loops were deleted before refinement and built in manually based on electron density maps. Illustrations and calculations of superpositioned models were prepared in PyMOL (The PyMOL Molecular Graphics System, Version 2.4.0 Schrödinger, LLC). Calculation of hinge relationships of domains was accomplished with HINGE (https://niaidsis.niaid.nih.gov/hinge.html), provided courtesy of Peter Sun, NIAID. Buried surface area (BSA) calculations were performed with PISA (https://www.ebi.ac.uk/pdbe/pisa/).

The final structures for the RBD-SB16 and RBD-SB45 complexes showed R_{work}/R_{free} (%) 25.4/27.7 and 18.6/21.6 respectively, and for SB16 alone with R_{work}/R_{free} 22.4/25.9. Data collection and structure refinement statistics are provided in Table S1.

bioRxiv preprint doi: https://doi.org/10.1101/2021.01.27.428466; this version posted January 27, 2021. The copyright holder for this preprint (which was not certified by peer review) is the author/funder. This article is a US Government work. It is not subject to copyright under 17 USC 105 and is also made available for use under a CC0 license.

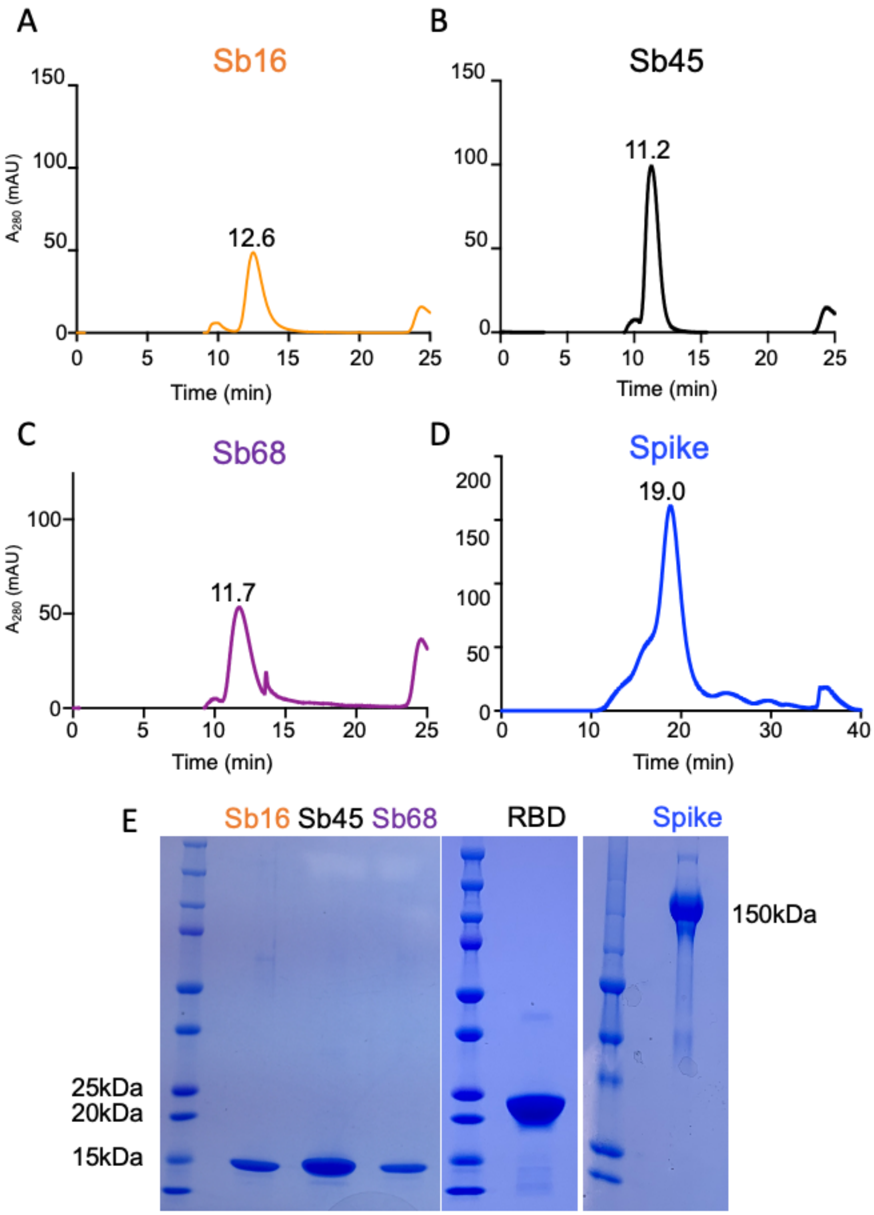

Figure S1.

Fig. S1. SEC profiles and purification of sybodies, RBD, and spike. (A, B, C) Monomeric sybodies, as indicated, were purified on SRT-10C-SEC100 columns. Elution time of each sybody is indicated above each peak. The *y* axis represents A_{280} nm absorbance units (mAu). (D) SEC profile of trimeric spike protein (Superose^{TM} 6 10/300 GL. (E) SDS-PAGE image of purified sybodies, RBD and spike protein.

bioRxiv preprint doi: https://doi.org/10.1101/2021.01.27.428466; this version posted January 27, 2021. The copyright holder for this preprint (which was not certified by peer review) is the author/funder. This article is a US Government work. It is not subject to copyright under 17 USC 105 and is also made available for use under a CC0 license.

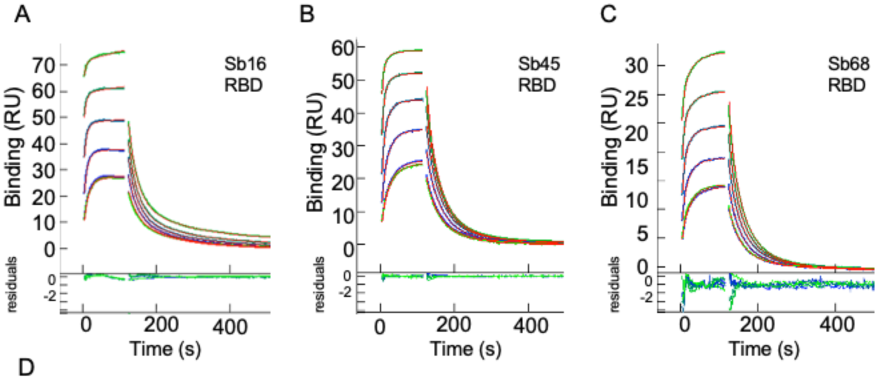

D

Sybody	K_{D1} (μM)	k_{off1}(s^{-1})	signal	K_{D2} (μM)	k_{off2}(s^{-1})	signal
Sb16	0.10 ± 0.02	0.05 ± <.001	38.0 ± 3.3	0.77 ± 0.035	0.002 ± 0.0002	31.5 ± 4.5
Sb45	0.038 ± .0005	0.03 ± 0.005	56.3 ± 13.5	0.69 ± 0.063	0.06 ± 0.001	23.5 ± 3.9
Sb68	0.041 ± .006	0.03 ± 0.001	23.5 ± 1.2	0.67 ± 0.021	0.039 ± 0.002	38.0 ± 1.15

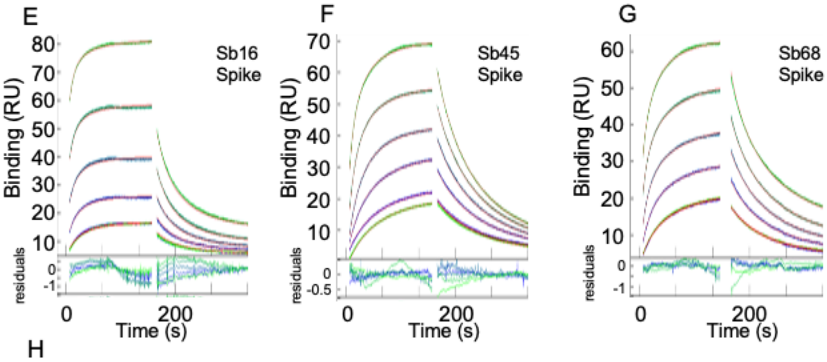

H

Sybody	K_{D1} (μM)	k_{off1}(s^{-1})	signal	K_{D2} (μM)	k_{off2}(s^{-1})	signal
Sb16	0.41 ± 0.12	0.05 ± 0.01	34.5 ± 10.1			
Sb45	0.82 ± 0.013	0.03 ± 0.01	27.7 ± 8.5	0.07 ± 0.01	0.016 ± 0.003	32.1 ± 10.4
Sb68	2.60± 1.4	0.049 ± 0.01	60.4± 12.8			

Figure S2.

Fig. S2. Sybodies bind RBD with K_D values in the nanomolar range. RBD (A, B, C) or S protein (E, F, G) was coupled to a biosensor chip as described in Materials and Methods. Graded concentrations (31 to 500 nM) were flowed over the coupled surfaces (from t=0 to t= 120 s, followed by buffer washout) and net RU signal (compared to mock-coupled surface) was measured by SPR for Sb16 (A, E); Sb45 (B, F); and Sb68 (C, G). Summary of triplicate mean ± SD determinations is shown in Tables (D, H). Global analysis (curve fits in red, residuals below the curves) was accomplished using EVILFIT (*37, 38*), and the major components of binding are shown.

bioRxiv preprint doi: https://doi.org/10.1101/2021.01.27.428466; this version posted January 27, 2021. The copyright holder for this preprint (which was not certified by peer review) is the author/funder. This article is a US Government work. It is not subject to copyright under 17 USC 105 and is also made available for use under a CC0 license.

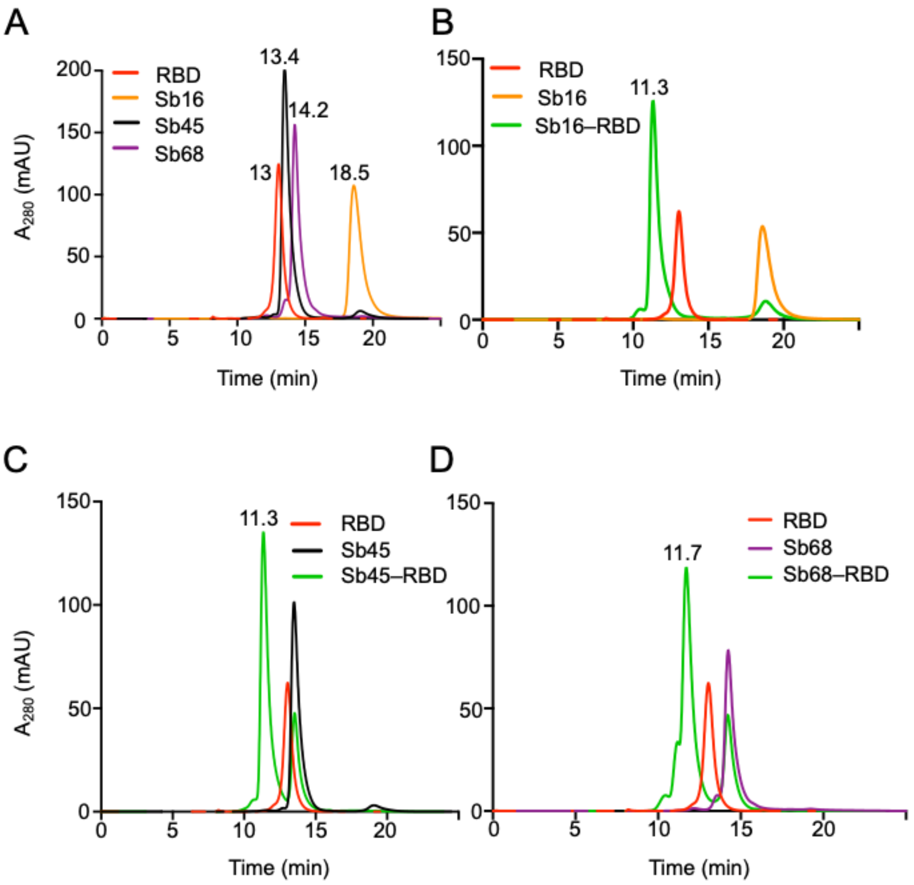

Figure S3.

Fig. S3. SEC profiles reveal direct interaction of sybodies with RBD. (A) Sybodies (50 µg) and RBD (50 µg) were injected onto a Shodex-KW-802.5 column (0.75 ml/min) in 100 µl individually and elution times are shown. Sb16 and RBD (B), Sb45 and RBD (C), or Sb68 and RBD (D) were mixed in equal concentrations (50 µg in 100 µl), incubated at 4 °C overnight and then analyzed on the Shodex column.

bioRxiv preprint doi: https://doi.org/10.1101/2021.01.27.428466; this version posted January 27, 2021. The copyright holder for this preprint (which was not certified by peer review) is the author/funder. This article is a US Government work. It is not subject to copyright under 17 USC 105 and is also made available for use under a CC0 license.

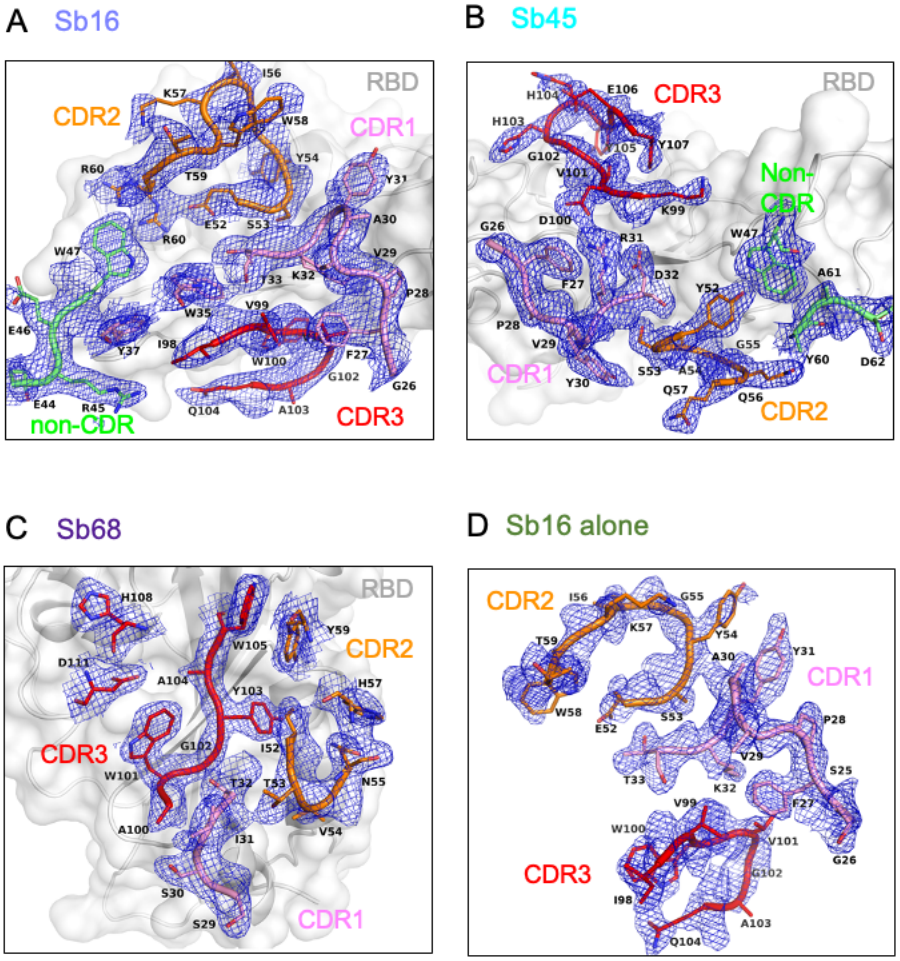

Figure S4.

Fig. S4. Electron density (as blue) maps (2mFo-DFc) for CDR loops and those residues in contact with RBD: (A) Sb16 on RBD surface, resolution=2.6 Å, R_{free}=0.277; (B) Sb45 on RBD surface, resolution=2.3 Å, R_{free}=0.216; (C) Sb68 on RBD surface, resolution=2.6 Å, R_{free}=0.255; (D) Sb16 alone, resolution=2.1 Å, R_{free}=0.259. Contour at 1.0σ, CDR1 loop as pink, CDR2 loop as orange, CDR3 loop as red, non-CDR residues as lime, and RBD is in background as gray.

bioRxiv preprint doi: https://doi.org/10.1101/2021.01.27.428466; this version posted January 27, 2021. The copyright holder for this preprint (which was not certified by peer review) is the author/funder. This article is a US Government work. It is not subject to copyright under 17 USC 105 and is also made available for use under a CC0 license.

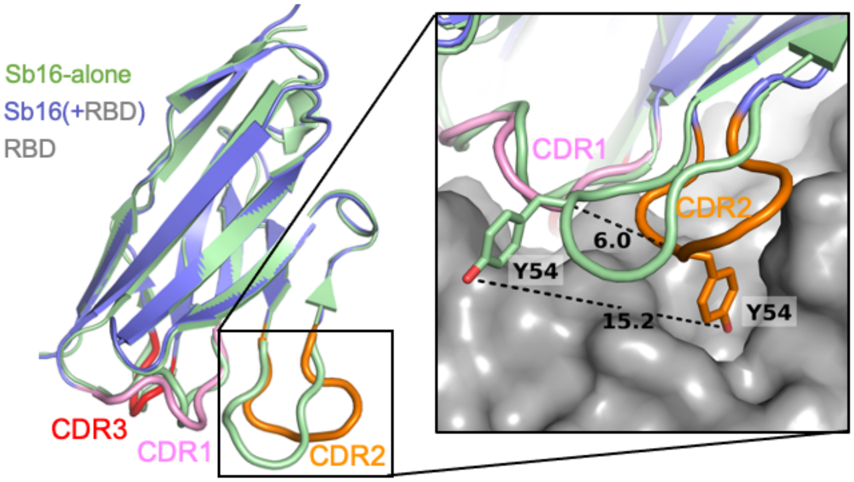

Figure S5.

Fig. S5. A superimposition of Sb16 alone ("unliganded", free, green) and Sb16–RBD ("liganded", complexed, slate) reveals the large movement of CDR2 loop (about 6 Å). Particularly, Y54 moved about 15 angstroms and dipped into a binding pocket which surrounded by epitopic residues of Q409, E406, D405, R403, G416, K417, I418, N422, L455, Y453, Y495. (RBD surface as gray).

bioRxiv preprint doi: https://doi.org/10.1101/2021.01.27.428466; this version posted January 27, 2021. The copyright holder for this preprint (which was not certified by peer review) is the author/funder. This article is a US Government work. It is not subject to copyright under 17 USC 105 and is also made available for use under a CC0 license.

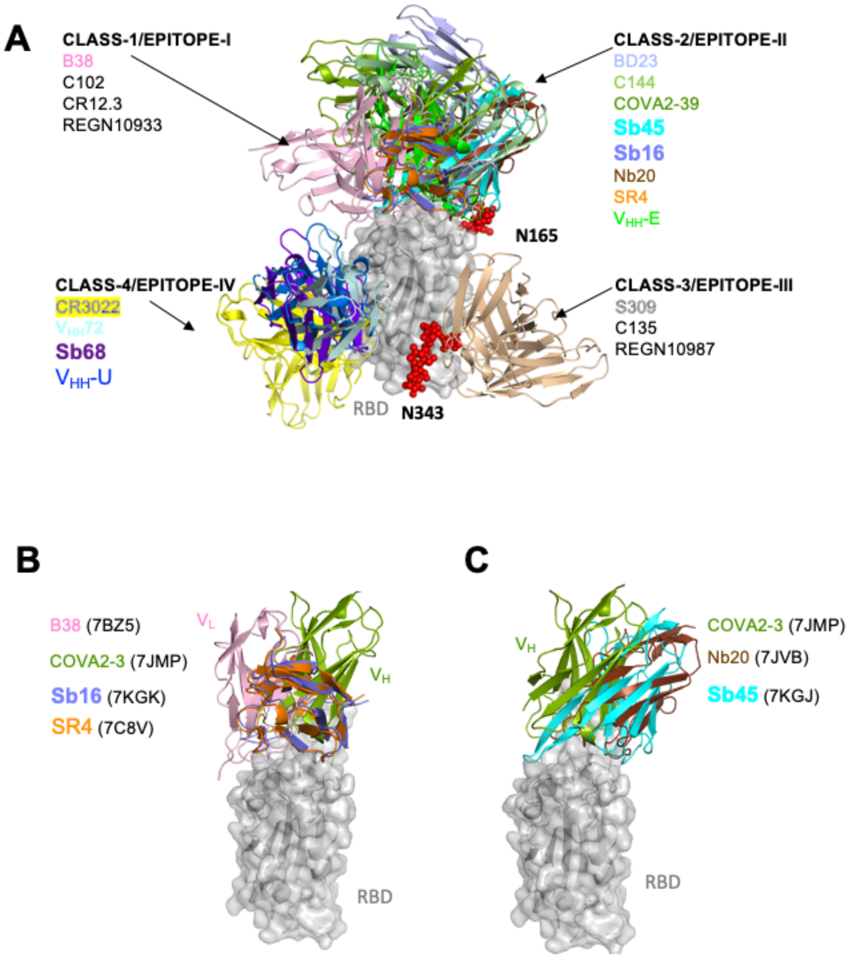

Figure S6

Fig. S6. Sybodies, nanobodies, and antibodies bind different epitopic regions of the RBD. (A) Definition of Classes according to Barnes et al. (*31*) and Epitope area according to Xiang et al.(*12*). Epitope I to IV are almost the same as Class 1 to 4, except for Epitope V which spans Class 3 and Class 4 (not shown). Color codes are for representative Fab (only shown are the variable domains) or sybody/nanobody. Sb68 falls into Class-4/Epitope-IV overlapping with V_{HH}72 and HH-E, and CR3022. RBD is in gray and two N-glycans (N165 and N343 in red) are shown. (B) Sb16 clashes with L-chain of B38 (7BZ5) of Class-1 and H-chain of COVA2-39 (7JMP) H-chain Class-2. It falls between Class-1/Epitope-I and Class-2/Epitope-II but almost

bioRxiv preprint doi: https://doi.org/10.1101/2021.01.27.428466; this version posted January 27, 2021. The copyright holder for this preprint (which was not certified by peer review) is the author/funder. This article is a US Government work. It is not subject to copyright under 17 USC 105 and is also made available for use under a CC0 license.

covers SR4. (C) Sb45 overlaps with H-chain of COVA2-39 (7JMP) of Class-2 and lies in the same orientation as Nb20 (7JVB) - Epitope-II.

bioRxiv preprint doi: https://doi.org/10.1101/2021.01.27.428466; this version posted January 27, 2021. The copyright holder for this preprint (which was not certified by peer review) is the author/funder. This article is a US Government work. It is not subject to copyright under 17 USC 105 and is also made available for use under a CC0 license.

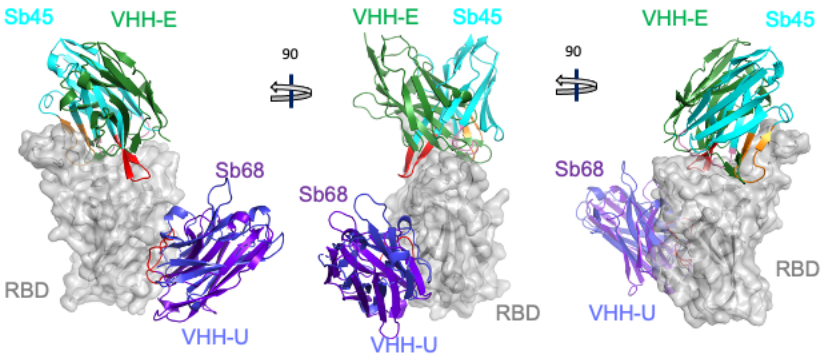

PDB	Interface		BSA($Å^2$)
7KLW	Sb45 = 1010	Sb68 = 640	total = 1650
7KN5	VHH-E = 821	VHH-U = 625	total = 1446

Figure S7

Fig. S7. Comparison of the ternary sybody structure (Sb45–RBD–Sb68, 7KLW) with the ternary nanobody structure (V_{HH}-E–RBD–V_{HH}-U, 7KN5), Koenig et al. (*32*). Sb45 in cyan, Sb68 in purpleblue, RBD in gray, V_{HH}-E in green, V_{HH}-U in blue. CDR1, CDR2, and CDR3 of Sb45, CDR3 of Sb68 are highlighted in pink, orange, and red. Three views are each rotated by 90°. CDR3 and CDR2 loops of Sb45 ride along both sides of the RBD, while V_{HH}-E uses only an extended CDR3 loop on the side. Sb68 is slightly lower than V_{HH}-U while V_{HH}-E is similar to V_{HH}72.

bioRxiv preprint doi: https://doi.org/10.1101/2021.01.27.428466; this version posted January 27, 2021. The copyright holder for this preprint (which was not certified by peer review) is the author/funder. This article is a US Government work. It is not subject to copyright under 17 USC 105 and is also made available for use under a CC0 license.

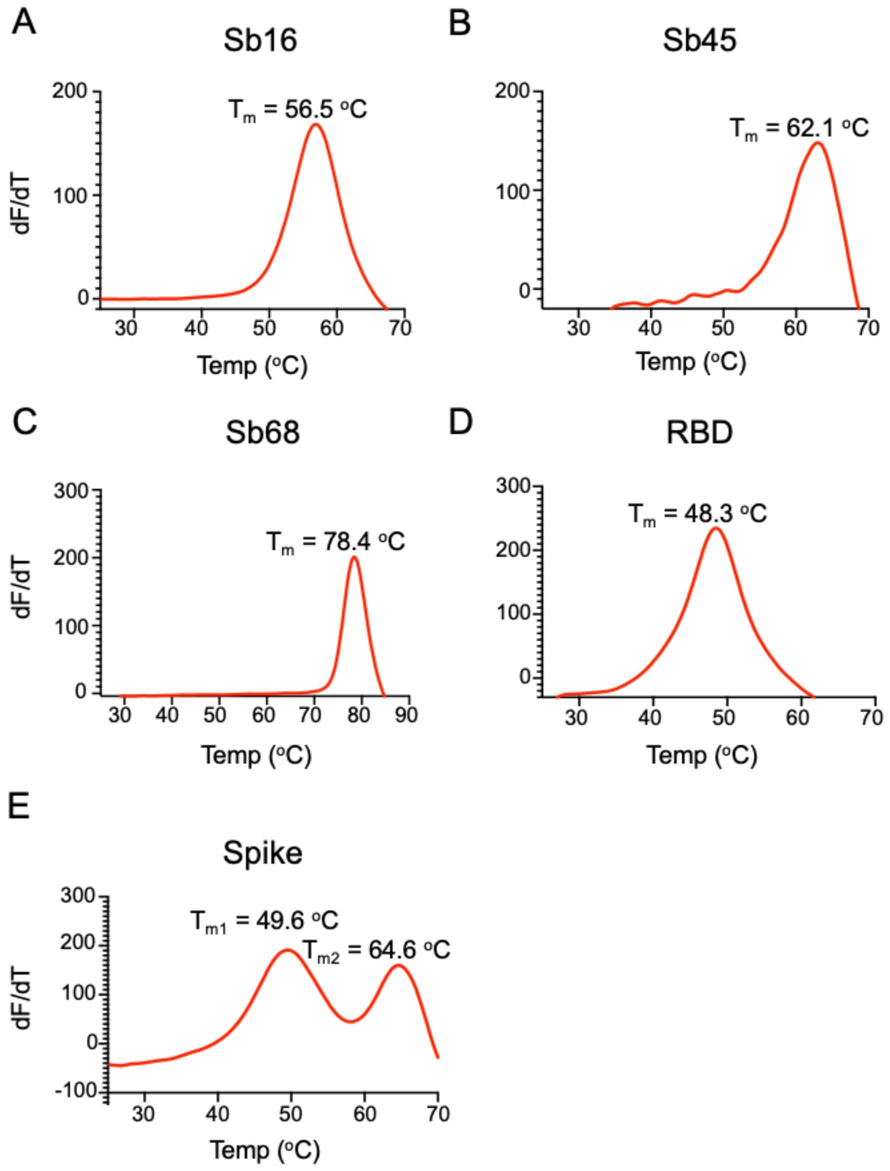

Fig. S8. Sybodies, RBD, and spike protein reveal unique thermal stability. T_m of each of the indicated purified proteins was determined by thermal melt analysis as described in Materials and Methods. Note the biphasic behavior of the trimeric S protein.

bioRxiv preprint doi: https://doi.org/10.1101/2021.01.27.428466; this version posted January 27, 2021. The copyright holder for this preprint (which was not certified by peer review) is the author/funder. This article is a US Government work. It is not subject to copyright under 17 USC 105 and is also made available for use under a CC0 license.

	**Sb16-RBD**	**Sb45-RBD**	**Sb45-RBD-Sb68**	**Sb16**
**PDBID**	**7KGK**	**7KGJ**	**7KLW**	**7KGL**
**Data collection**				
Space group	$P6_522$	$P3_221$	$C222_1$	$P6_322$
Cell dimensions				
a, *b*, *c* (Å)*	65.64, 65.64, 344.69	62.55, 62.55, 168.82	74.50, 102.40, 138.97	69.32, 69.32, 105.57
α, *β*, *γ* (°)□	90.0, 90.0, 120.0	90.0, 90.0, 120.0	90.0, 90.0, 90.0	90.0, 90.0, 120.0
Resolution (Å)	57.34-2.60 (2.69-2.60)	45.59-2.30 (2.38-2.30)	44.12-2.60 (2.69-2.60)	60.0-2.10 (2.18-2.10)
R_{sym} or R_{merge}	0.080 (0.455)	0.101 (0.849)	0.095 (0.739)	0.055 (0.714)
$I/\sigma(I)$	18.0 (3.3)	14.9 (3.4)	13.1 (2.1)	17.8 (2.7)
Completeness (%)	98.8 (99.1)	99.3 (98.3)	98.8 (98.7)	98.9 (98.4)
Redundancy	10.3 (10.9)	7.9 (8.2)	7.2 (7.4)	6.1 (6.5)
R_{pim}	0.024 (0.134)	0.038 (0.293)	0.038 (0.287)	0.025 (0.311)
$CC_{1/2}$	0.999 (0.987)	0.997 (0.919)	0.998 (0.895)	0.999 (0.891)
Estimated twin fraction	0.0 (none)	0.06 (-h, -k, l)	0.0 (none)	0.0 (none)
**Refinement**				
Resolution (Å)	56.09-2.60 (2.69-2.60)	45.59-2.30 (2.38-2.30)	36.72-2.60 (2.69-2.60)	32.9-2.10 (2.18-2.10)
No. reflections	13219 (1185)	17592 (1687)	16508 (1627)	9278 (823)
R_{work}/R_{free} (%)	25.8/27.7 (36.3/44.2)	18.6/21.6 (24.1/29.8)	20.6/25.5 (29.3/34.5)	22.4/25.9 (31.9/31.4)
No. atoms	2486	2641	3552	980
Protein	2486	2500	3456	913
Water + ligands	0	141	96	67
B-factor Wilson/Average	39.3/59.8	26.9/32.9	33.9/31.5	23.2/36.9
Protein	59.8	32.8	31.5	37.0
Water + ligands	0	34.7	29.5	35.0
R.m.s. deviations				
bond length (Å)	0.002	0.005	0.003	0.003
bond angle (º)	0.54	0.74	0.64	0.57
Ramachandran				

bioRxiv preprint doi: https://doi.org/10.1101/2021.01.27.428466; this version posted January 27, 2021. The copyright holder for this preprint (which was not certified by peer review) is the author/funder. This article is a US Government work. It is not subject to copyright under 17 USC 105 and is also made available for use under a CC0 license.

favored (%)	92.9	97.4	96.3	93.0
allowed (%)	7.1	2.6	3.7	6.1
outliers (%)	0.0	0.0	0.0	0.9

Table S1. X-ray data collection and refinement statistics
*Values in parenthesis are for highest resolution shell.

bioRxiv preprint doi: https://doi.org/10.1101/2021.01.27.428466; this version posted January 27, 2021. The copyright holder for this preprint (which was not certified by peer review) is the author/funder. This article is a US Government work. It is not subject to copyright under 17 USC 105 and is also made available for use under a CC0 license.

ACE2+RBD (6M0J)

RBD	ACE2	DIST	ACE2	DIST
K417	D30	2.90		
Y449	Q42	2.79	D38	2.70
Y453	H34	2.86		
L455	H34	3.62		
F456	T27	3.49		
A475	Q24	3.65	T27	3.97
G476	Q24	4.43		
F486	Y83	3.52	M82	3.64
N487	Y83	2.79	Q24	2.69
Y489	F28	3.55	Y83	3.55
Q493	K31	2.93	E35	3.13
G496	K31	3.08		
Q498	Q42	2.93	Y41	3.59
T500	R357	3.51	N330	3.64
N501	K353	3.61		
G502	K353	2.78		
Y505	E37	3.46	K353	3.61

Sb45+RBD (7KGJ)

RBD	Sb45	DIST	Sb45	DIST	CDR
Y351	A54	3.80			CDR2
R403	H103	3.00	G102	3.30	CDR3
G446	F27	3.70	G26	3.90	CDR1
G447	P28	3.50			CDR1
Y449	D100	3.80	R31	3.20	CDR3
N450	Y30	3.50			CDR1
L452	S53	3.90			CDR2
Y453	V101	3.40			CDR3
T470	G55	3.80	R59	2.95	CDR2
I472	R59	3.90			
G482	R59	3.30			
V483	Y60	3.60	D62	4.10	
E484	R33	2.50	Y52	2.60	CDR1
Y489	K99	4.20			CDR3
F490	A54	3.70			CDR2
L492	A54	3.70			CDR2
Q493	K99	3.10	D32	3.41	CDR3
S494	D32	2.80			CDR1
Y495	V101	3.80			CDR3
Q498	G26	3.30			CDR1
N501	H103	3.50			CDR3
G502	H103	3.30			CDR3
Y505	V101	2.86	H103	3.80	CDR3

Sb16+RBD (7KGK)

RBD	Sb16	DIST	Sb16	DIST	CDR
R403	Y54	3.00			CDR2
E406	Y54	2.70			CDR2
K417	Y54	3.30			CDR2
V445	E44	3.00			
G446	Y37	3.30			CDR1
G447	Y37	3.20			CDR1
Y449	I98	3.60	Q104	3.70	CDR3
L452	W100	3.29			CDR3
Y453	S53	2.95	Y54	3.50	CDR2
L455	Y31	3.49	S53	3.44	CDR1
F456	Y31	3.37			CDR1
E484	K32	2.39	F27	3.30	CDR1
G485	P28	3.38			CDR1
Y489	Y31	3.41	A30	3.34	CDR1
F490	W100	3.38			CDR3
L492	W100	3.85			CDR3
Q493	K32	3.51	T33	3.70	CDR1
S494	T33	3.73			CDR1
Y495	W35	3.13	Y54	4.30	CDR2
G496	W35	3.71			CDR1
Q498	Y37	2.91	W47	3.48	
N501	R60	2.38			
G502	R60	3.60			
Y505	A57	3.50	E52	3.80	

Sb68+RBD (7KLW)

RBD	Sb68	DIST	Sb68	DIST	CDR
Y369	T32	3.30	Y103	3.50	CDR1
N370	N55	3.30	V54	3.90	CDR2
S371	N55	4.40			CDR2
A372	H57	3.20			CDR2
F374	Y103	2.90	Y59	3.40	CDR3
S375	A104	3.40	W105	2.80	CDR3
T376	A104	3.40	Y103		CDR3
F377	G102	3.40	Y103	2.90	CDR3
K378	D111	3.30	W101	3.40	CDR3
C379	W101	2.80			CDR3
V382	S29	4.30			CDR1
S383	A100	4.40			CDR3
P384	G101	3.60	A100	3.60	CDR3
T385	T32	3.70			CDR1
R408	H108	3.20	D111	2.60	CDR3

Table S2A. Interactions between RBD Residues and either ACE2 or sybody residues. In each rectangle (first column) is listed the identification of an RBD amino acid in contact with one or two residues of the indicted chain. Distance between residues is given in Å. For the sybodies, the particular CDR designation is also indicated.

bioRxiv preprint doi: https://doi.org/10.1101/2021.01.27.428466; this version posted January 27, 2021. The copyright holder for this preprint (which was not certified by peer review) is the author/funder. This article is a US Government work. It is not subject to copyright under 17 USC 105 and is also made available for use under a CC0 license.

```
        370       380       390       400       410       420
        |         |         |         |         |         |
RBD    YNSASFSTFKCYGVSPTKLNDLCFTNVYADSFVIRGDEVRQIAPGQTGKIADYNYKLPDDF
ACE2                                                   *
Sb16                                     *  *          *
Sb45
Sb68   **** ******   ***                      *
---------------------------------------------------------------------
       445  450       460       470       480       490       500
       |    |         |         |         |         |         |
RBD    VGGNYNYLYRLFRKSNLKPFERDISTEIYQAGSTPCNGVEGFNCYFPLQSYGFQPTNGVGY
ACE2      *  *   * **                   *          ** *   *  * ***  *
Sb16   *** *  ** *                               **   ** ***** *  **  *
Sb45    ** ** **                 * *          ***    ** ****  *  **  *
```

229 **Table S2B.** Tabulation of residues of RBD that contact each of the indicated chains, based on 6M0J for ACE2–RBD, 7KGK for Sb16–RBD, 7KGJ for Sb45–RBD, and 7KLW for the Sb68–RBD interface. Note that ACE2 contacts broadly overlap those for Sb16 and Sb45, but that Sb68 contacts are distinct.

bioRxiv preprint doi: https://doi.org/10.1101/2021.01.27.428466; this version posted January 27, 2021. The copyright holder for this preprint (which was not certified by peer review) is the author/funder. This article is a US Government work. It is not subject to copyright under 17 USC 105 and is also made available for use under a CC0 license.

**Complex (A + B)**	**BSA($Å^2$)**	$_A N_{res}$	$_B N_{res}$	N_{HB}	N_{SB}	**PDB**	**Res. Å**
ACE2+RBD	**844**	26	26	13	2	6M0J	2.45
Sb16+RBD	**1003**	26	29	8	1	7KGK	2.60
Sb45+RBD	**976**	27	33	15	4	7KGJ	2.10
*(Sb45)+RBD	**1010**	26	35	14	4	7KLW_B	2.60
*(Sb68)+RBD	**640**	21	17	9	4	7KLW_C	2.60
*(Sb45+Sb68)+RBD	**1650**	47	52	23	8	7KLW	2.60
*(VHH-E)+RBD	**821**	29	27	13	1	7KN5_C	1.87
*(VHH-U)+RBD	**625**	16	20	17	0	7KN5_E	1.87
*(VHH-E+VHH-U)+RBD	**1446**	45	47	30	1	7KN5	1.87
H11D4+RBD	**599**	20	20	11	4	6YZ5	1.80
H11H4+RBD	**637**	17	19	5	4	6ZBP	1.85
(CR3022)+RBD	**991**	19	20	7	4	6XC7_HL	2.88
VHH72+RBD	**796**	21	25	9	2	6WAQ_A	2.20
Nb20+RBD	**705**	22	21	9	4	7JVB	3.29
Nb6+Spike	**788**	24	21	9	1	7KKK_D	3.03
Sb23+Spike	**772**	21	22	5	0	7A25_D	3.06
Sb23+Spike	**585**	18	21	5	0	7A29_D	2.94
Ty1+Spike	**795**	23	26	3	0	6ZXN_D	2.93
C144+Spike	**689**	22	24	7	0	7K90_H	3.24
C002+Spike	**728**	22	23	7	1	7K8S_H	3.40
*(REGN10933)+RBD	**935**	28	30	9	1	6XDG_BD	3.90
*(REGN10987)+RBD	**607**	21	19	5	0	6XDG_AC	3.90

bioRxiv preprint doi: https://doi.org/10.1101/2021.01.27.428466; this version posted January 27, 2021. The copyright holder for this preprint (which was not certified by peer review) is the author/funder. This article is a US Government work. It is not subject to copyright under 17 USC 105 and is also made available for use under a CC0 license.

*(REGN10933+ REGN10987)+RBD	**1542**	49	49	5	0	6XDG	3.90

Table S3. Variety of Buried Surface Area (BSA) at interfaces of Sybodies, Nanobodies, and Fabs with RBD or Spike. BSA of each of indicated interface was calculated from the relevant chains using PISA (https://www.ebi.ac.uk/pdbe/pisa/), to account for the non-overlapping surface area. BSA in Å^2. N_{res} indicates the number of residues in contact; N_{HB}, the number of potential hydrogen bonds; N_{SB}, the number of potential salt bridges.
